# Mapping multicenter randomized controlled trials in anesthesiology: a scoping review

**DOI:** 10.1186/s13643-021-01776-5

**Published:** 2021-10-26

**Authors:** Sylvain Boet, Joseph K. Burns, Olivia Cheng-Boivin, Hira Khan, Kendra Derry, Deric Diep, Abdul Hadi Djokhdem, Sung Wook Um, Johnny W. Huang, Danica Paré, Mimi Deng, Liza Begunova, Linda Yi Ning Fei, Maryam Bezzahou, Pium Sonali Andrahennadi, Elysia Grose, Ruth G Abebe, Fadi Mansour, Zoé Talbot, Pierre-Marc Dion, Manvinder Kaur, Justen Choueiry, Cole Etherington

**Affiliations:** 1 Department of Anesthesiology and Pain Medicine, University of Ottawa, 451 Smyth Rd, Ottawa, ON K1H 8L1 Canada; 2 Clinical Epidemiology Program, Ottawa Hospital Research Institute, 501 Smyth Rd, Ottawa, ON K1H 8L6 Canada; 3 Department of Innovation in Medical Education, University of Ottawa, 451 Smyth Rd, Ottawa, ON K1H 8L1 Canada; 4 Faculty of Medicine, University of Ottawa, 451 Smyth Rd, Ottawa, ON K1H 8L1 Canada; 5 Department of Health Sciences, Carleton University, 1125 Colonel By Drive, Ottawa, ON K1S 5B6 Canada; 6 Department of Anesthesiology and Pain Medicine, University of Toronto, Toronto, Canada; 7 Department of Psychological and Brain Sciences, Dartmouth College, Hanover, NH 03755 USA; 8 Faculty of Health Sciences, University of Ottawa, 125 University, Ottawa, ON K1N 6N5 Canada; 9 Intensive Care Unit, Montfort Hospital, Ottawa, Canada

**Keywords:** Anesthesiology, Randomized controlled trials, Multicenter trials, Scoping review

## Abstract

**Background:**

Evidence suggests that there are substantial inconsistencies in the practice of anesthesia. There has not yet been a comprehensive summary of the anesthesia literature that can guide future knowledge translation interventions to move evidence into practice. As the first step toward identifying the most promising interventions for systematic implementation in anesthesia practice, this scoping review of multicentre RCTs aimed to explore and map the existing literature investigating perioperative anesthesia-related interventions and clinical patient outcomes.

**Methods:**

Multicenter randomized controlled trials were eligible for inclusion if they involved a tested anesthesia-related intervention administered to adult surgical patients (≥ 16 years old), with a control group receiving either another anesthesia intervention or no intervention at all. The electronic databases Embase (via OVID), MEDLINE, and MEDLINE in Process (via OVID), and Cochrane Central Register of Control Trials (CENTRAL) were searched from inception to February 26, 2021. Studies were screened and data were extracted by pairs of independent reviewers in duplicate with disagreements resolved through consensus or a third reviewer. Data were summarized narratively.

**Results:**

We included 638 multicentre randomized controlled trials (*n* patients = 615,907) that met the eligibility criteria. The most commonly identified anesthesia-related intervention theme across all studies was pharmacotherapy (*n* studies = 361 [56.6%]; *n* patients = 244,610 [39.7%]), followed by anesthetic technique (*n* studies = 80 [12.5%], *n* patients = 48,455 [7.9%]). Interventions were most often implemented intraoperatively (*n* studies = 233 [36.5%]; *n* patients = 175,974 [28.6%]). Studies typically involved multiple types of surgeries (*n* studies = 187 [29.2%]; *n* patients = 206 667 [33.5%]), followed by general surgery only (*n* studies = 115 [18.1%]; *n* patients = 201,028 [32.6%]) and orthopedic surgery only (*n* studies = 94 [14.7%]; *n* patients = 34,575 [5.6%]). Functional status was the most commonly investigated outcome (*n* studies = 272), followed by patient experience (*n* studies = 168), and mortality (*n* studies = 153).

**Conclusions:**

This scoping review provides a map of multicenter RCTs in anesthesia which can be used to optimize future research endeavors in the field. Specifically, we have identified key knowledge gaps in anesthesia that require further systematic assessment, as well as areas where additional research would likely not add value. These findings provide the foundation for streamlining knowledge translation in anesthesia in order to reduce practice variation and enhance patient outcomes.

**Supplementary Information:**

The online version contains supplementary material available at 10.1186/s13643-021-01776-5.

## Background

Each year around the world, over four million people die within 30 days of surgery [1]. Care provided or organized by anesthesiologists throughout the perioperative period is critical to surgical patient outcomes [2, 3]; yet, practice variation is widespread [4–9]. Despite the need to increase adherence to evidence-based practice in anesthesia, there has not yet been a comprehensive summary of the anesthesia literature that can guide future knowledge translation, i.e., the synthesis, dissemination, and uptake of research evidence into practice [10, 11].

After systematic reviews, randomized controlled trials (RCTs) are considered the strongest level of evidence in medicine. RCTs often recruit participants from multiple centers to assess intervention effectiveness across a variety of settings (i.e., external validity) and/or to obtain sufficient statistical power when studying outcomes with a low incidence rate (e.g., mortality) [12, 13]. Although single-center RCTs may be large and powerful, summarizing multicenter RCTs in anesthesia is an important step toward identifying the most promising interventions for systematic implementation in anesthesia practice across a wide range of patient outcomes.

Scoping reviews map key concepts and types of evidence available for complex research areas that have not been comprehensively and systematically reviewed before [14–16]. Due to the broad nature of perioperative and anesthesia research, a scoping review is needed to provide the foundation for future systematic reviews and KT to move evidence into practice [14, 17]. Mapping anesthesia-related interventions (i.e., interventions performed, organized, or initiated during the perioperative period by a healthcare professional with specific training in anesthesia) and clinical patient outcomes investigated in multicentre RCTs can inform future practice change initiatives. Specifically, a scoping review can inform the development of targeted systematic review questions focusing on particular areas of interest [18]. As the anesthesia literature currently stands, it is unclear where saturation has been reached and where knowledge gaps remain.

This scoping review of multicenter RCTs aims to systematically explore and map the existing literature investigating perioperative anesthesia-related interventions on patient outcomes. This work may help to guide priorities for KT in anesthesiology as well as for future research.

## Methods

The PRISMA Extension for Scoping Reviews (PRISMA-ScR) Checklist [19] guided the conduct and reporting of this scoping review.

### Eligibility criteria

We selected studies if they involved a tested anesthesia-related intervention administered to adult surgical patients (≥ 16 years old), with a control group receiving either another anesthesia intervention or no intervention at all. We defined anesthesia-related interventions as “interventions provided in the perioperative period that either were or could have been, performed, organized, or initiated by a healthcare professional with specific training in anesthesia” [20]. The perioperative period was defined as the time period beginning 24 h before the surgical procedure to 24 h following the procedure. We excluded studies involving surgical procedures with only local anesthesia. We included all multicenter randomized controlled trials (i.e., trials involving two or more centers) assessing the impact of anesthesia interventions on one or more patient outcomes. We elected to only include multicentre randomized controlled trials to ensure impactful resource allocation, minimization of research duplication, and potentially better research coordination.

### Information sources and search strategy

We searched the electronic databases Embase (via OVID), MEDLINE, and MEDLINE in Process (via OVID), and Cochrane Central Register of Control Trials (CENTRAL). A separate search strategy was constructed for each database, reviewed by the research team, and refined as necessary (see Appendix). The MEDLINE search strategy was reviewed by a second trained information scientist as per Peer Review of Electronic Search Strategies (PRESS) guidelines [21–23]. Clinical trial registries and reference lists of included studies and previously published systematic reviews were also searched. Date and language restrictions were not imposed for the literature search; however, only studies reported in English were included in the scoping review. The search included studies published up until February 26, 2021.

### Selection of sources of evidence

Studies were selected by seven pairs of independent reviewers using DistillerSR (Evidence Partners, Ottawa, Canada), a web-based systematic review software. A screening tool featuring questions based on inclusion and exclusion criteria was developed, piloted, and refined as necessary. Reviewer calibration occurred during this process, whereby screeners clarified questions and reasons for decision-making until satisfactory inter-rater reliability was achieved (kappa > 0.60). Seven pairs of independent reviewers conducted eligibility screening of titles and abstracts in duplicate. Studies were “excluded” at this stage if the two independent reviewers determined they did not meet eligibility criteria. Otherwise, studies proceeded to full-text screening. Disagreements about inclusion or exclusion at each stage were resolved by consensus or through a third member of the research team (NE, OCB) as needed. The final list of included articles was reviewed by the investigator team to determine if any additional articles should have been included. The citations of the excluded articles are provided in Additional file 2.

### Data charting process

Seven pairs of independent reviewers conducted data extraction using an electronic form (DistillerSR, Evidence Partners, Ottawa, Canada) created and piloted by the research team. The following information was extracted: publication details (e.g., first author name, publication year, country of data collection), study details (e.g., design and sample size), patient demographics (e.g., sex, age, coexisting medical conditions), intervention details (e.g., type, duration), comparator (i.e., no intervention or other anesthesia intervention), and patient outcome (e.g., outcome definition and timing). Accuracy was compared and verified by the reviewer pairs upon completion of data extraction. Quality assessments are typically not completed for scoping reviews and were therefore not conducted [17, 24].

### Synthesis of results

Anesthesia-related interventions were classified according to themes developed in a previous scoping review [20]. Similar outcomes were grouped into larger categories.

The results of this scoping review were summarized using a narrative approach, along with a quantitative summary of relevant study characteristics.

### Ethical approval

The design of this study did not require the approval of an ethics committee as it is a scoping review. However, the research team did raise and consider the ethical aspects surrounding this study prior to data collection.

## Results

**Fig. 1:**
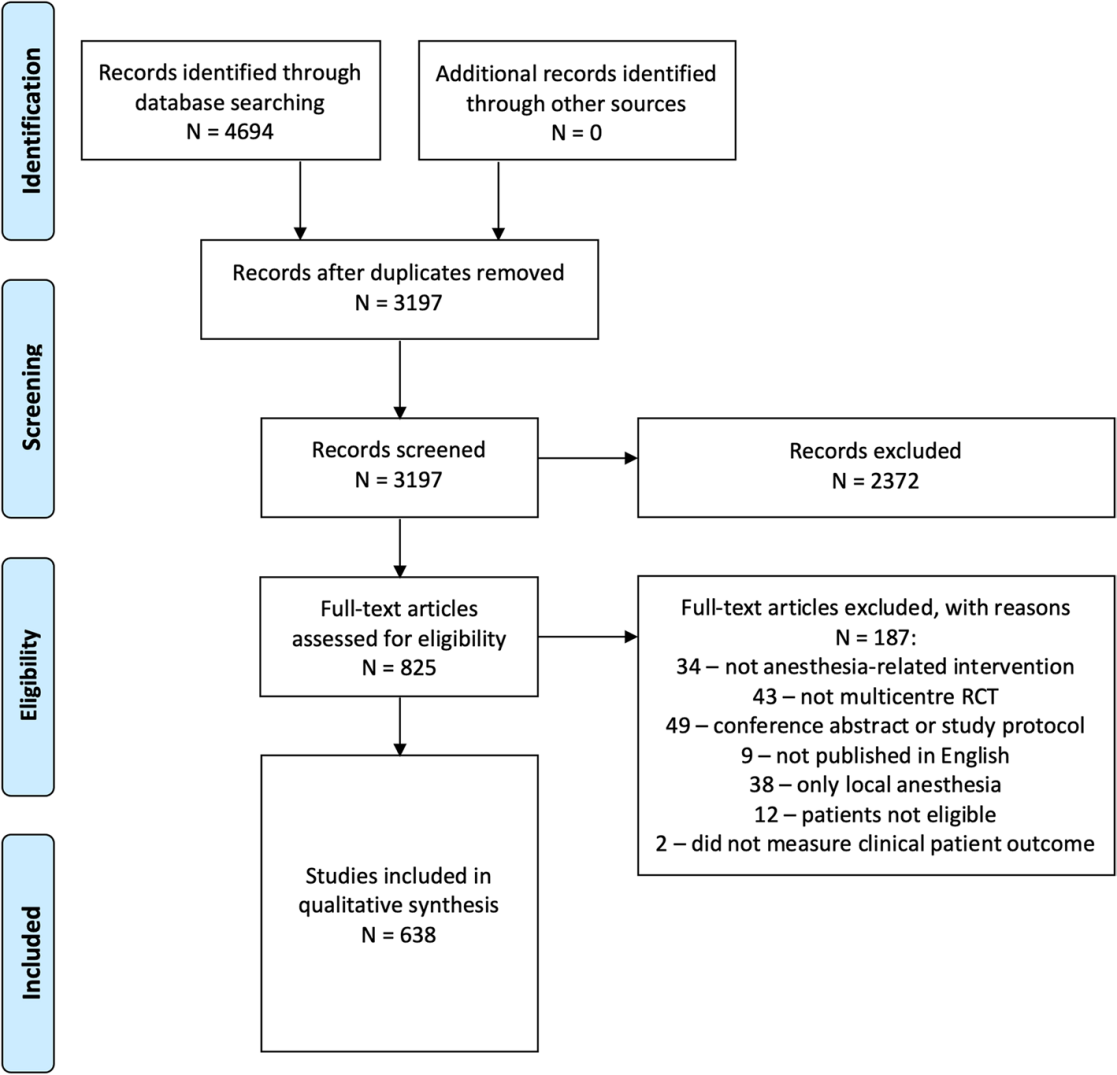
PRISMA flow chart

The literature search retrieved 4694 publications. After removal of duplicates, 3197 articles proceeded to the screening process. Following title and abstract screening for inclusion, 2372 articles were excluded. Full-text review for inclusion/exclusion criteria led to the exclusion of another 187 articles. A final total of 638 articles were therefore included in this scoping review (Fig. 1).

**Fig. 2:**
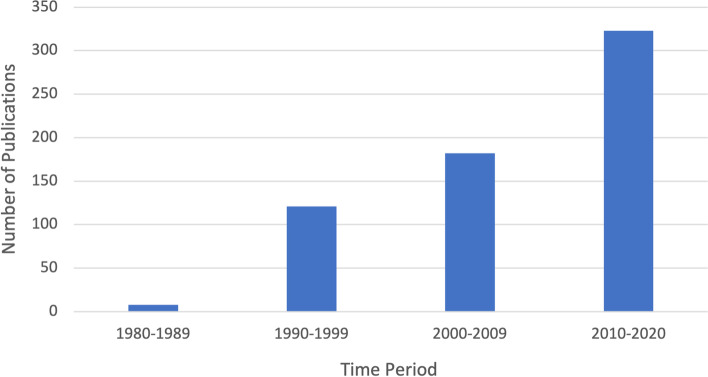
Evolution of the number of multicenter randomized controlled trials in anesthesia

A total of 615,907 participants were randomized across all 638 trials. Publication of multicenter trials began after the year 1980 and was highest in the years 2010–2020 (Fig. 2), although the time period extending beyond the search data appears poised to supersede the previous decade. Data collection occurred most often in the USA (*n* studies = 165 [25.9%]; *n* patients = 102,063 [16.8%]) (Supplemental Fig. 1). The median number of centers involved in a single study was 11 (IQR = 6–18).

**Fig. 3:**
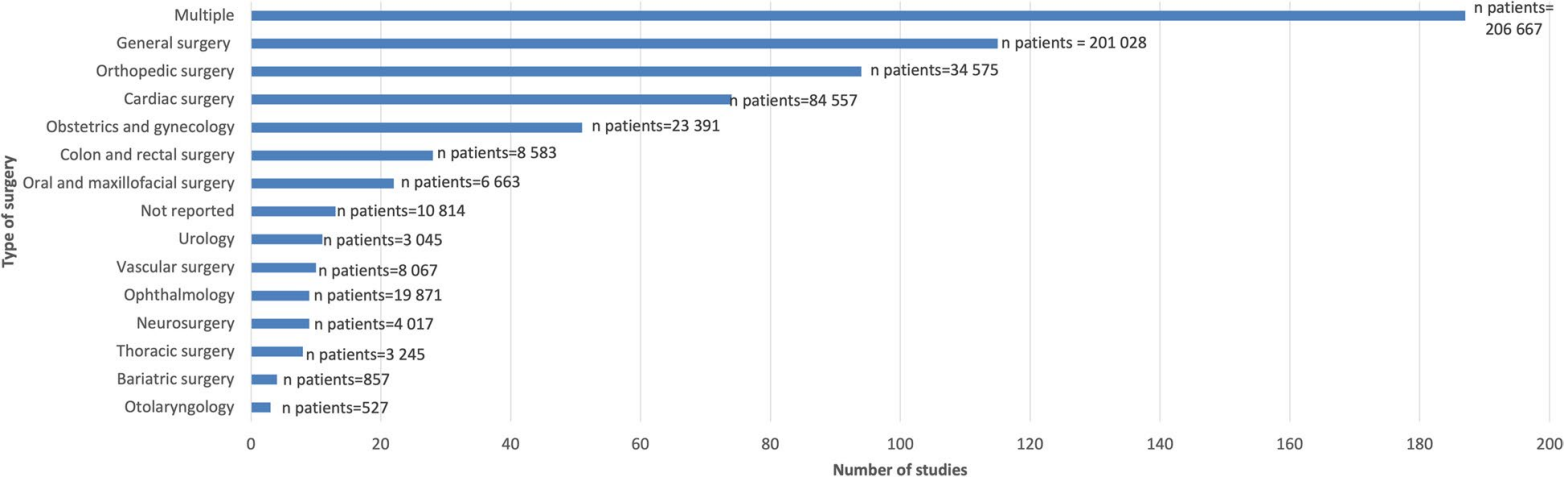
Type of surgery involved in multicenter randomized controlled trials of anesthesia-related interventions

The most commonly identified anesthesia-related intervention theme across all multicentre RCTs by study number was pharmacotherapy (*n* studies = 361 [56.6%]; *n* patients = 244 610 [39.7%]), followed by anesthetic technique (*n* studies = 80 [12.5%]; *n* patients = 48 455 [7.9%]) (Table 1). Interventions were most often implemented intraoperatively alone (*n* studies = 233 [36.5%]; *n* patients = 175 974 [28.6%]) as opposed to pre- and/or postoperatively (Table 2). As shown in Fig. 3, multicenter RCTs typically involved several types of surgery (*n* studies = 187 [29.3%]; *n* patients = 206 667 [33.6%]), followed by general surgery only (*n* studies = 115 [18.0%]; *n* patients = 201,028 [32.6%]) and orthopedic surgery only (*n* studies = 94 [14.7%]; *n* patients = 34,575 [5.6%]). Less than 10 multicenter RCTs investigated anesthesia-related interventions within the context of bariatric surgery, neurosurgery, ophthalmology, otolaryngology, thoracic surgery, urology, and vascular surgery.

**Table 1:** Anesthesia-related intervention themes in multicenter randomized controlled trials (*n* studies = 445, *n* patients = 336 966)

**Intervention category**	**Number of studies (%)**	**Number of patients (%)**
Anesthetic technique	80 (12.5)	48 455 (7.9)
Behavioral change	4 (0.7)	438 (0.07)
Dialysis	0 (0)	0 (0)
Glucose control	0 (0)	0 (0)
Intravenous (IV) fluids	12 (1.9)	3 671 (0.5)
Medical device	6 (1.1)	1 166 (0.1)
Monitoring	11 (1.7)	15 912 (2.6)
Nutritional	20 (3.1)	4 731 (0.7)
Pharmacotherapy	361 (56.6)	244 610 (39.7)
Physiotherapy	4 (0.7)	994 (0.2)
Preoperative procedure	18 (2.8)	9904 (1.6)
Protocol/guidelines implementation	12 (1.8)	159 134 (25.8)
Temperature management	5 (0.8)	2 451 (0.4)
Testing	0 (0)	0 (0)
Transfusion	19 (3.0)	13 994 (2.3)
Ventilation	19 (3.0)	11 817 (1.9)
Combination of interventions	67 (10.5)	98 641 (16.0)
**Total**	638	615 907

This list of themes was generated in our previously conducted scoping review [20]

**Table 2:** Perioperative phase of anesthesia-related interventions in multicenter randomized controlled trials (*n* studies = 445, *n* patients = 336 966)

Perioperative phase	Number of studies (%)	Number of patients (%)
Preoperative	67 (10.5)	47 702 (7.7)
Intraoperative	233 (36.5)	175 974 (28.6)
Postoperative	165 (25.9)	209 743 (34.1)
Multi-phase (i.e., intervention spanned across 2 or 3 phases)	169 (26.5)	179 650 (29.2)
Not reported	4 (0.6)	2630 (0.4)
**Total**	638	615 907

A summary of patient outcomes investigated by the included studies is displayed in Table 3. Outcomes reported here include both primary and secondary outcomes. Of note, studies often investigated more than one outcome and were therefore counted more than once. Functional status was the most commonly investigated outcome according to its number of studies (*n* studies = 272), followed by patient experience (*n* studies = 168) and mortality (*n* studies = 153). Studies investigating mortality involved the greatest number of patients (*n* patients = 412,416), followed by those investigating length of stay (*n* patients = 283,463), and cardiovascular outcomes (*n* patients = 276,808).

**Table 3:** Outcomes investigated by anesthesia multicentre randomized controlled trials (*n* = 445)

**Outcome category**	**Number of studies**	**Number of patients**
Blood clot/bleeding	73	60 969
Cardiovascular	131	276 808
Functional status	272	95 202
Gastrointestinal	38	47 996
Hematological	57	67 258
Infection	102	141 554
Injury or damage to tissue or organ	28	24 325
Length of stay	142	283 463
Mortality	153	412 416
Neurological	55	130 369
Patient experience	168	83 598
Psychiatric-related outcome	29	27 570
Pulmonary	92	208 132
Renal	60	67 354
Wound	26	30 224
Other	166	261 629

## Discussion

This scoping review identified 638 multicenter RCTs investigating the impact of anesthesia-related interventions on clinical patient outcomes. Most RCTs took place in the USA and investigated pharmacotherapy interventions implemented intraoperatively. Functional status was the most commonly investigated outcome, followed by patient experience and mortality. By mapping the anesthesia literature at a high level of evidence, such as multicenter RCTs, this review can be used to guide future research and intervention development with the potential to optimize surgical patient care and outcomes. Specifically, we have identified a clear under-investigation of non-pharmacotherapy interventions in the anesthesia literature. Future RCTs and systematic reviews may therefore consider how anesthesia-related interventions outside of pharmacotherapy impact patient outcomes (e.g., teamwork interventions). Knowledge translation interventions can then be developed to move this evidence into clinical practice and to standardize the use of non-pharmacotherapy interventions when they may benefit patients the most. The aim of a scoping review is to map evidence in a field rather than to conduct a thorough analysis of each included study. Accordingly, quality of evidence was not assessed and results should be interpreted accordingly [14].

We observed a clear gap in the literature, for example, related to behavior change or nutritional interventions, which were investigated by a limited number of studies. This finding is similar to a previous scoping review on anesthesia-related interventions that specifically analyzed mortality, including both single and multicenter trials [20]. Unlike the previous review, however, we did not limit our work to a specific type of surgery (e.g., emergency only [25]) and included a wide range of clinical patient outcomes beyond mortality. It is surprising that studies frequently investigated functional status and patient experience outcomes but tended to focus on provider-centric interventions (e.g., pharmacotherapy). Other scoping reviews have also focused on provider-centric interventions such as mode of anesthesia [25], with limited discussion of interventions that may provide patients with a greater sense of control over their care and outcomes. With both surgical patients [26, 27] and anesthesia providers [28, 29] increasingly interested in alternative therapies, perhaps more investigation is needed regarding non-pharmacotherapy interventions. This may provide opportunities for enhancing patient-centered care and advancing anesthesia practice.

It is noteworthy that patient-centered outcomes were among the most commonly investigated by multicenter trials within the anesthesia literature given recent calls to systematically incorporate these outcomes into perioperative research and practice (e.g., decision-making, surgical care plans) [30]. Many surgical specialties have moved toward developing standardized patient-centered endpoints [31–33]. Based on this review, there appears to be a strong foundation for anesthesiology to move toward doing the same. For example, over 450 studies included in this review examined how anesthesia-related interventions impact patient experience (e.g., patient satisfaction, patient evaluation of care), length of stay, psychiatric-related outcomes, or patient functional status (e.g., health-related quality of life, pain, mobility, quality of recovery). Recently, Jerath and colleagues validated days alive and out of hospital as a patient-centered outcome for perioperative medicine [34]. Of course, this is just one potential patient-centered outcome to consider amidst the several highlighted by our review.

Our review also highlights that anesthesia-related interventions implemented in the intra- or post-operative period are the most studied by multicenter trials. However, only 67 studies investigated interventions implemented preoperatively. This may suggest a key opportunity regarding both the existence and effectiveness of preventative interventions. There may be a need to further explore preoperative optimization of surgical patients given the persistent rate of complications that still occur despite advancements in anesthetic and surgical care [35, 36]. For example, future multicenter trials may consider investigating nutritional or physical exercise interventions in the preoperative period to optimize patient outcome [37]. Another important consideration for preventing postoperative complications may be the role of teamwork and communication. Research suggests that ineffective teamwork in the OR is a primary contributing factor in two out of three cases of postoperative complications [38]; however, we did not identify any multicenter study examining teamwork-related interventions. This is a significant knowledge gap for the perioperative community to address. In addition, most of the included studies were conducted in North America or Europe, yet surgical and anesthesia complications are even more prevalent in other parts of the world [1]. Given the lack of studies from low- and middle-income countries, there is an urgent need for more research in these contexts in order to reduce the overall global burden of anesthesia-related morbidity and mortality.

Anesthesia-related interventions were typically examined within the context of multiple types of surgery, orthopedic surgery only, or general surgery only. These interventions appear to be less investigated within particular surgical specialties, such as bariatric surgery, neurosurgery, ophthalmology, otolaryngology, thoracic surgery, urology, and vascular surgery. Future multicenter trials may therefore aim to test effectiveness of anesthesia-related interventions within different surgical specialties.

### Strengths and limitations

There are several limitations of this scoping review. The breadth and volume of the included studies prevented further assessment of intervention effectiveness. It will be important for future systematic reviews to examine the effectiveness of specific intervention themes for particular outcome categories. Our scoping review provides a useful map for this purpose given its extensive summary of a very large number of studies investigating a diverse range of interventions and patient outcomes. For example, future systematic reviews may also wish to quantify the effect of non-pharmacotherapy interventions compared to pharmacotherapy interventions for specific patient-centered patient outcomes. Our review also included only those studies published in English for feasibility reasons. Given our aim was to provide a map of the literature rather than to summarize treatment effects, we believe that inclusion of studies published in other languages would change our results. With the map we have provided, researchers can conduct more targeted systematic reviews in the future. Within a narrower review, inclusion of non-English studies would be less resource-intensive. Ultimately, our scoping review is an important step toward improving the practice of anesthesia and benefited from a comprehensive search strategy, clear inclusion and exclusion criteria, and significant content expertise among our team of co-investigators. Another limitation is our narrow eligible perioperative period of 24 h before and after the surgical procedure to 24 h following the procedure. It is likely that a wider time window would result in additional interventions taking place in the days before or after surgery, such as prehabilitation with exercise therapy.

## Conclusion

This scoping review provides a map of multicenter RCTs in anesthesia which can be used to optimize future research endeavors in the field. Specifically, we have identified key knowledge gaps in anesthesia that require further systematic assessment, as well as areas where additional research would likely not add value. These findings provide the foundation for streamlining knowledge translation in anesthesia in order to reduce practice variation and enhance patient outcomes.

### Supplementary Information

**Additional file 1: Supplemental Fig. 1.** Country of data collected for anesthesia-related interventions tested in multicentre randomized controlled trials.

**Additional file 2.**

## Data Availability

The data for this study are available upon request from the study team.

## Appendix

### Literature search strategy

#### MEDLINE search strategy

1. exp Surgical Procedures, Operative/
2. ((operat* or surger* or surgical*) adj3 an?esth*).tw.
3. (surger* or surgical*).tw.
4. or/1–3
5. exp Postoperative Period/
6. exp Perioperative Care/
7. Preoperative Care/
8. exp Anesthesia/
9. exp Analgesia/
10. ((perioperative or intraoperative or postoperative or peroperative) adj3 (care or therap* or treatment* or procedure* or management*)).tw.
11. an?esth*.tw.
12. analges*.tw.
13. or/5–12
14. exp Morbidity/
15. exp Postoperative Complications/
16. Mortality/
17. Survival Rate/
18. Hospital Mortality/
19. Mortality, Premature/
20. morbid*.tw.
21. mortal*.tw.
22. (survivo?r* adj3 (rate* or time*)).tw.
23. survivo?rship*.tw.
24. (premature adj3 death*).tw.
25. (death adj2 rate*).tw.
26. fatalit*.tw.
27. exitus.tw.
28. or/14–27
29. clinical trial, phase i/ or clinical trial, phase ii/ or clinical trial, phase iii/ or clinical trial, phase iv/ or controlled clinical trial/ or exp randomized controlled trial/
30. exp Clinical Trials as Topic/
31. Double-Blind Method/
32. Single-Blind Method/
33. Random Allocation/
34. Clinical Trial/
35. clinical trial*.tw.
36. (singl* or doubl* or trebl* or tripl*).tw.
37. 35 or 36
38. (mask* or blind*).tw.
39. 37 and 38
40. randomized controlled trial.pt.
41. controlled clinical trial.pt.
42. clinical trial.pt.
43. random*.tw.
44. control*.tw.
45. 29 or 30 or 31 or 32 or 33 or 34 or 39 or 40 or 41 or 42 or 43 or 44
46. Multicenter Studies as Topic/
47. multicenter study.pt.
48. multicenter study/
49. multi?cent*.tw.
50. 46 or 47 or 48 or 49
51. 45 and 50
52. 4 and 13 and 28 and 51

#### Embase search strategy

1. surgical technique/
2. ((operat* or surger* or surgical*) adj3 an?esth*).ti,ab.
3. (surger* or surgical*).ti,ab.
4. or/1–3
5. postanesthesia care/ or postoperative analgesia/ or postoperative care/
6. perioperative period/
7. preoperative period/ or preoperative care/
8. anesthesia/
9. ((perioperative or intraoperative or postoperative or peroperative) adj3 (care or therap* or treatment* or procedure* or management*)).tw.
10. postoperative analgesia/
11. or/5–10
12. morbidity/
13. postoperative complication/
14. mortality/
15. survival rate/
16. morbid*.tw.
17. mortal*.tw.
18. or/12–17
19. clinical trial/
20. “clinical trial (topic)”/ or exp “controlled clinical trial (topic)”/ or “phase 1 clinical trial (topic)”/ or “phase 2 clinical trial (topic)”/ or “phase 3 clinical trial (topic)”/ or “phase 4 clinical trial (topic)”/
21. exp controlled clinical trial/ or phase 1 clinical trial/ or phase 2 clinical trial/ or phase 3 clinical trial/ or phase 4 clinical trial/
22. or/19–21
23. “multicenter study (topic)”/
24. multicenter study/
25. or/23–24
26. 4 and 11 and 18 and 22 and 25

## Abbreviations

KT: Knowledge translation
RCT: Randomized controlled trial
