# Correction to: Mapping multicenter randomized controlled trials in anesthesiology: a scoping review

**DOI:** 10.1186/s13643-022-01899-3

**Published:** 2022-02-09

**Authors:** Sylvain Boet, Joseph K. Burns, Olivia Cheng-Boivin, Hira Khan, Kendra Derry, Deric Diep, Abdul Hadi Djokhdem, Sung Wook Um, Johnny W. Huang, Danica Paré, Mimi Deng, Liza Begunova, Linda Yi Ning Fei, Maryam Bezzahou, Pium Sonali Andrahennadi, Elysia Grose, Ruth G. Abebe, Fadi Mansour, Zoé Talbot, Pierre-Marc Dion, Manvinder Kaur, Justen Choueiry, Cole Etherington

**Affiliations:** 1 Department of Anesthesiology and Pain Medicine, University of Ottawa, 451 Smyth Rd, Ottawa, ON K1H 8L1 Canada; 2 Clinical Epidemiology Program, Ottawa Hospital Research Institute, 501 Smyth Rd, Ottawa, ON K1H 8L6 Canada; 3 Department of Innovation in Medical Education, University of Ottawa, 451 Smyth Rd, Ottawa, ON K1H 8L1 Canada; 4 Faculty of Medicine, University of Ottawa, 451 Smyth Rd, Ottawa, ON K1H 8L1 Canada; 5 Department of Health Sciences, Carleton University, 1125 Colonel By Drive, Ottawa, ON K1S 5B6 Canada; 6 Department of Anesthesiology and Pain Medicine, University of Toronto, Toronto, Canada; 7 Department of Psychological and Brain Sciences, Dartmouth College, Hanover, NH 03755 USA; 8 Faculty of Health Sciences, University of Ottawa, 125 University, Ottawa, ON K1N 6N5 Canada; 9 Intensive Care Unit, Montfort Hospital, Ottawa, Canada

**Correction to: Syst Rev 10, 276 (2021)**

10.1186/s13643-021-01776-5

Following publication of the original article [1], the 22^nd^ author was omitted from the author group. Justen Choueiry has been added to the author group and is presented correctly in this correction article.

The original article [1] has been corrected.
