# Supplementary figures and images for: Mapping multicenter randomized controlled trials in anesthesiology: a scoping review
Source: Syst Rev. 2021 Oct 26;10:276. doi: 10.1186/s13643-021-01776-5 (PMC8549299; doi:10.1186/s13643-021-01776-5)

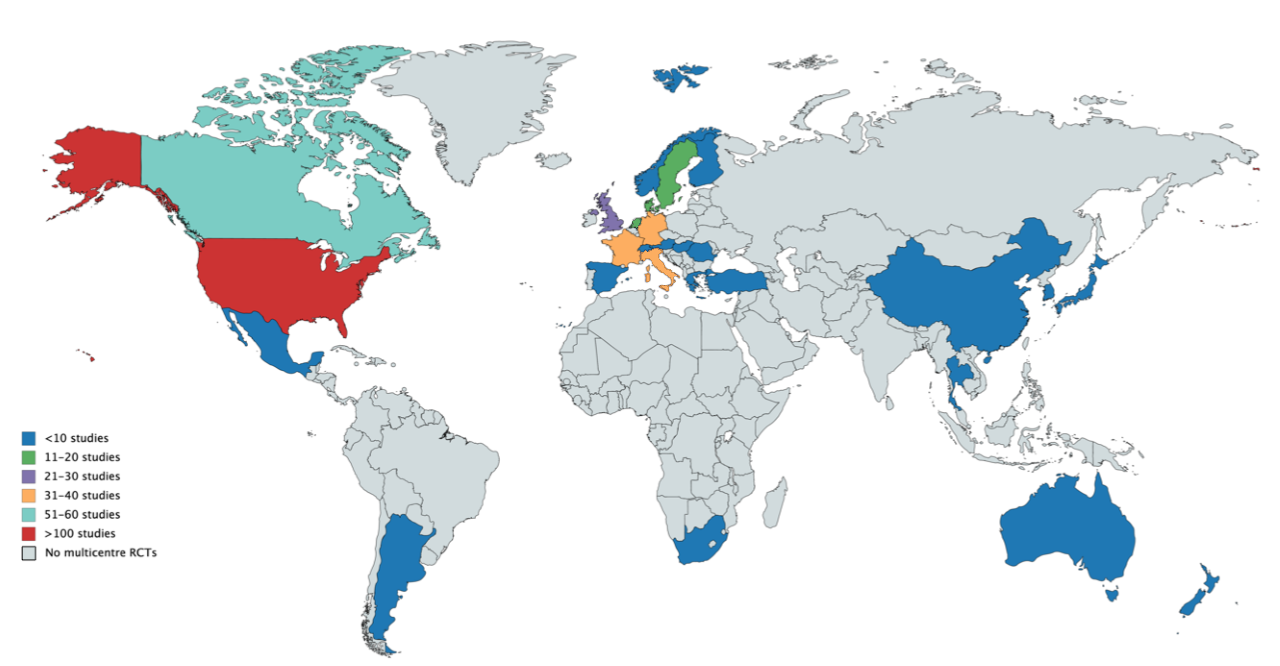

Supplement: Supplementary file 1 — Additional file 1: Supplemental Fig. 1. Country of data collected for anesthesia-related interventions tested in multicentre randomized controlled trials. [file 13643_2021_1776_MOESM1_ESM.tiff]
